# Meta-barcoding in combination with palynological inference is a potent diagnostic marker for honey floral composition

**DOI:** 10.1186/s13568-017-0429-7

**Published:** 2017-06-24

**Authors:** Rama Chandra Laha, Surajit De Mandal, Lalhmanghai Ralte, Laldinfeli Ralte, Nachimuthu Senthil Kumar, Guruswami Gurusubramanian, Ramalingam Satishkumar, Raja Mugasimangalam, Nagesh Aswathnarayana Kuravadi

**Affiliations:** 1 Departments of Botany, Biotechnology and Zoology, School of Life Sciences, Mizoram University, Aizawl, Mizoram 796004 India; 2 Department of Biotechnology, Bharathiar University, Coimbatore, 641046 India; 3 Genotypic Technologies, Bangalore, India; 4 QTLomics Technologies, Bangalore, India

**Keywords:** Honey, Multilocus target, DNA barcoding, Palynology

## Abstract

Identification of floral samples present in honey is important in order to determine the medicinal value, enhance the production of honey as well as to conserve the honey bees. Traditional approaches for studying pollen samples are based on microscopic observation which is laborious, time intensive and requires specialized palynological knowledge. Present study compares two composite honey metagenome collected from 20 samples in Mizoram, Northeast India using three gene loci- rbcL, matK and ITS2 that was sequenced using a next-generation sequencing (NGS) platform (Illumina Miseq). Furthermore, a classical palynology study for all 20 samples was carried out to evaluate the NGS approach. NGS based approach and pollen microscopic studies were able to detect the most abundant floral components of honey. We investigated the plants that were frequently used by honey bees by examining the results obtained from both the techniques. Microscopic examination of pollens detected plants with a broad taxonomic range covering 26 families. NGS based multigene approach revealed diverse plant species, which was higher than in any other previously reported techniques using a single locus. Frequently found herbaceous species were from the family Poaceae, Myrtaceae, Fabaceae and Asteraceae. The future NGS based approach using multi-loci target, with the help of an improved and robust plant database, can be a potential replacement technique for tedious microscopic studies to identify the polleniferous plants.

**Electronic supplementary material:**

The online version of this article (doi:10.1186/s13568-017-0429-7) contains supplementary material, which is available to authorized users.

## Introduction

Honey has been used for centuries as a complex natural sweetener having therapeutic properties. Bees obtain pollen and nectar from flowers and hence the plant composition varies due to different topography, climate and farming practices. The knowledge of flora in a region is essential for successful bee keeping, management of bee colonies and production of other bee products.

Conventional methods to analyze the association between plants and pollinators depend on time intensive observation of individual interaction (Mitchell et al. 2009). Few methods have been proposed for the determination of botanical and geographical origin of honey. The conventional approach used microscopic observation of pollens present in honey (palynology), which is very tedious and time consuming process. The other common chemical methods based on aroma compounds, free amino acids or minerals and trace element were also developed, but requires sophisticated and expensive instruments (Hermosín et al. 2003; Fernández-Torres et al. 2005; Anklam et al. 1998). Moreover, all these methods provide only limited information on the plant composition of honey samples. While some efforts have been made to develop protocols to ascertain the entomological sources of honey (Schnell et al. 2010), most have focused on identifying its plant origin. Past studies have often relied upon diagnostic phytochemicals (Cotte et al. 2004; Tosun 2013) or the study of pollen in honey (melissopalynology) (Alves and Santos 2014). Although the latter approach requires considerable expertise and cannot distinguish many plant species (Kaškonienė and Venskutonis 2010), yet it is a powerful diagnostic tool, especially when used with other methods (Hawkins et al. 2015). However, melissopalynology is ineffective in cases where low value honey is filtered to remove its source pollen and spiked with pollen from the desired monoflora (Kaškonienė and Venskutonis 2010).

With the advancement in next generation sequencing technology, study of the botanical and geographical origin of honey is much easier since it is fast, precise and reliable. Both Roche 454 and Illumina sequencing has been successfully used in analysing mixed species in various applications. Metabarcoding, identification of genera or species present in a composite DNA sample has been introduced by Richardson et al. (2015a) targeting the ITS2 marker using Illumina sequencing technology. This also had higher sensitivity and resolution in identification of plant species than microscopic analysis of the pollen samples. Ion Torrent platform was used to evaluate the DNA barcoding technology for quantifying airborne pollen (Richardson et al. 2015b), whereas pyrosequencing was also successfully used to analyse pollen from honey samples (Sickel et al. 2015; Keller et al. 2015).

Here, we evaluated the botanical composition of honey samples to verify the hypothesis that the metabarcoding will reveal more information congruent with the palynological study. To test this multigene Illumina approach along with microscopic observation were used.

## Materials and methods

### Study site and honey sampling

Twenty different honey samples were collected from Aizawl and Champhai districts of Mizoram, an eastern Himalayan biodiversity hotspot, Northeast India. The honey samples were obtained from bee keepers during February to June 2014 (Table 1). Mizoram state is situated in the extreme end of the Himalayan ranges and is predominantly mountainous terrain. The region enjoys a moderate climate, tropical location and due to its high elevation with an annual average rainfall of 250 cm.

**Table 1:** Geographical location of the honey samples used in this study

District	Sample ID	Sample location	Latitude	Longitude	District	Sample ID	Sample location	Latitude	Longitude
Aizawl	A1	Falkland	23.73°	92.74°	Champhai	C1	Hmunhmeltha	23.4°	93.2°
	A2	Thuampui	23.74°	92.73°		C2	N.Khawbung	23.54°	93.31°
	A3	Tanhril	23.74°	92.67°		C3	Ruantlang	23.44°	93.34°
	A4	Durtlang	23.79°	92.72°		C4	Zote	23.49°	93.35°
	A5	Sihphir	23.81°	92.73°		C5	Khawzawl	23.37°	93.12°
	A6	Hlimen	23.77°	92.66°		C6	Vengsang	23.47°	93.31°
	A7	Melthum	23.69°	92.72°		C7	Chawngtlai	23.44°	93.19°
	A8	Maubawk	23.72°	92.69°		C8	Tlangsam	23.46°	93.34°
	A9	Sairang	23.80°	92.65°		C9	N.Champhai	23.45°	93.32°
	A10	Sakawrtuichhun	23.76°	92.67°		C10	Mualkawi	23.41°	93.33°

### Palynological study

#### Preparation of pollen slides from honey: acetolysis method

One millilitre of honey sample was taken in a test tube and diluted to 10 ml by hot distilled water of 40 °C. The diluted honey was sieved through a mesh of 100 µm. The suspension thus obtained was centrifuged at 3000 rpm for 5 min. The pellet of pollen sediment was subjected for acetolysis (Louveaux et al. 1978). Pollen grains were examined and identified under the light microscope. Percentage occurrence of pollen was used to determine their frequencies for determining the major and minor honeybee plants. Fresh flower of known plant pollen slides was prepared according to same acetolysis method as reference for identification (Louveaux et al. 1978).

#### Pollen spectrum study

The pollen grains were identified using local flora and confirmed by comparing pollen types with reference pollen slides. Based on the frequencies of pollen grain in various honey samples, the pollen count and percentage of pollen types were calculated and pollen spectra were prepared (Erdtman 1960). These pollen types were classified based on the recommendation of the International Commission for bee-Botany: “secondary pollen type (S)” (16–45%), “important minor pollen type (I)” (3–15%) and “minor pollen type (M)” (<3%).

#### Preparation of honey for DNA extraction

Honey samples were dissolved in 1 ml sterile water, incubated at 65 °C for 30 min followed by centrifugation at 5000 rpm for 10 min. The supernatant was discarded, and the pellet was dried for 5 min at room temperature and further dissolved in 500 µl extraction buffers (100 mM Tris–HCl, 50 mM EDTA, 50 mM NaCl, 10% SDS, pH 7.5). 0.5 g of sterilized glass beads (0.5–1 mm diameter) was added and the pellet was ground with a glass rod for 5–10 min. 100 µl DTT (110 mM) and 10 µl proteinase K (10 mg/ml) were added to the mixture and incubated at 56 °C for 1 h. A second incubation (65 °C for overnight) was performed by adding 500 µl cetyltrimethyl ammonium bromide (CTAB) extraction buffer (20 mM Tris–HCl, pH 8.0, 10 mM EDTA, pH 8.0, 10% CTAB, 5% polyvinylpyrrolidone), 10 µl proteinase K, and 50 µl DTT. Phenol–chloroform–isoamyl alcohol (500 µl) was added and centrifuged at 10,000 rpm for 10 min. DNA was precipitated using 500 µl isopropanol and 100 µl sodium acetate (3 mM) (Lalhmangaihi et al. 2014). The extracted DNA was checked by agarose gel electrophoresis and stored at −20 °C prior to subsequent analysis.

#### Amplification of the DNA barcode genes

For Illumina sequencing, all ten honey DNA samples from each district were pooled to make a composite DNA sample (Chp = composite DNA sample from Champhai district and Azl = composite honey DNA sample from Aizawl district). DNA from the two composite honey DNA samples (Chp and Azl) was amplified using three candidate DNA barcode gene primers: matK, rbcL and ITS2 (Table 2) (18, 19). PCR was performed in a total of 50 µl reaction volume consisting of 50 ng of DNA, 1X PCR buffer (75 mM Tris–HCl (pH 9.0), 50 mM KCl, 20 mM (NH4)_2_SO_4_), 2.5 mM MgCl_2_, 0.125 mM of each dNTPs, 0.5 µM of each primer and 0.5 U of Taq Polymerase (3B DNA polymerase, 3B Black Bio Biotech India). All PCR reactions was performed in an Agilent Sure Cycler 8800 using a touchdown amplification profile consisting of an initial denaturation at 95 °C for 5 min followed by 40 cycles of denaturation at 95 °C for 2 min, annealing at 65 °C for 90 s, extension at 72 °C for 2 min with a final extension at 72 °C for 10 min. In this touchdown protocol, the annealing temperature was uniformly decreased from 65 °C to 45 °C at the rate of 1 °C per cycle. The PCR products were resolved using 2% Agarose gel at 120 V till the samples reached 3/4th of the gel. The gel was visualized under UV light and the image was captured. The PCR products from each sample with the three primers was pooled in an equal concentration and preceded for NGS sequencing.

**Table 2:** Primers used for PCR amplification

Locus	Primer name	Direction	Sequence (5′–3′)
matK	KIM3	Forward	CGTACAGTACTTTTGTGTTTACGAG
	KIM1	Reverse	ACCCAGTCCATCTGGAAATCTTGGTTC
rbcL	rbcLa	Forward	ATGTCACCACAAACAGAGACTAAAGC
	rbcLajf634R	Reverse	GAAACGGTCTCTCCAACGCAT
ITS2	ITS2_3	Forward	GCATCGATGAAGAACGCAGC
	ITS2_4	Reverse	TCCTCCGCTTATTGATATGC

### Illumina sequencing

**Fig. 1:**
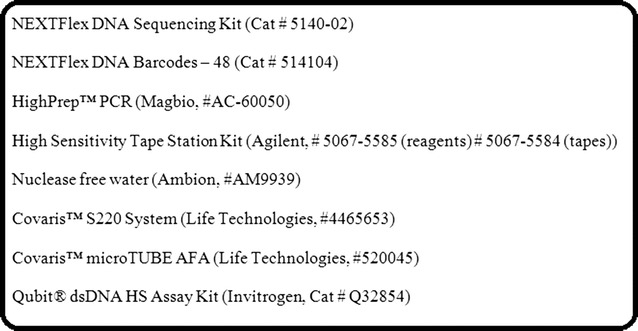
NEXTFlex DNA sample preparation guide. Illumina-compatible libraries were prepared using the above procedure

An Illumina-compatible library was prepared at Genotypic Technology, Bangalore, India according to manufacturer recommended protocol (Fig. 1). In brief, pooled amplicons were sheared to generate fragments of approximately 200–500 bp in a Covaris micro tube with the E220 system (Covaris, Inc., Woburn, MA, USA). The fragment size distribution was confirmed with Agilent High Sensitivity DNA Tape station (Agilent Technologies, Santa Clara, CA). Next, the fragmented DNA was cleaned up using HighPrep beads (MagBio Genomics, Inc, Gaithersburg, Maryland) followed by end-repair, A-tailing, and ligation of the Illumina multiplexing adapters. The adapter-ligated DNA was cleaned up using HighPrep beads (MagBio Genomics, Inc, Gaithersburg, Maryland). Then, the adapter ligated fragments were subjected to 10 rounds of PCR (denaturation at 98 °C for 2 min, cycling (98 °C for 30 s, 65 °C for 30 s and 72 °C for 1 min) and final extension at 72 °C for 5 min) and the amplicons were purified with HighPrep beads. The Illumina-compatible libraries were quantified with Qubit flourometer and their fragment length distribution was analyzed on Agilent High Sensitivity DNA Tape station (Agilent Technologies, California, USA). The Illumina sequencing was carried out using Illumina Nextseq 500 platform.

### Data analysis

The Illumina raw reads were quality checked using Fast QC followed by adapter clipping and trimming of low quality bases trimming towards 3′-end using fastx toolkit (Andrews and Fast 2010; Martin 2011; Gordon and Hannon 2010). De novo assemblies of quality filtered reads were carried out using velvet assembler (Zerbino and Birney 2008). The kmer value was optimized to select the best kmer for the assembly. The contigs were analysed by BLAST against NCBI Viridiplantae database to annotate the sequences in the assembly (Altschul et al. 1990). The annotation results were analysed and removed any duplicates to identify the species present in the sample.

## Results

### Pollen spectrum analysis of honey samples

Polleniferous plants were classified based on nature of vegetation such as wild plants, horticultural plants, ornamental plants and agricultural plants. Under wild plants, 42 species were identified majority of them taxonomically belongs to the family *Fabaceae* followed by *Asteraceae, Euphorbiaceae, Malvaceae, Myrtaceae, Lamiaceae, Rosaceae*, *Combretaceae*, *Verbenaceae*, *Betulaceae*, *Polygonaceae*, *Amaranthaceae*, *Oxalidaceae*, *Bombacaceae*, *Fagaceae*, *Rubiacea*e, *Cyperaceae*, *Elaeocarpaceae*, *Lythraceae*, *Solanaceae* and *Bignoniaceae*. Eleven species falls under the horticultural plants taxonomically classified under the family *Myrtaceae*, *Caricaceae*, *Rutaceae*, *Arecaceae*, *Rubiaceae*, *Lythraceae*, *Anacardiaceae*, *Musaceae* and *Vitaceae*. Ornamental plants consist of 8 species, major two falls under the family *Malvaceae* followed by each from *Asteraceae*, *Rubiaceae*, *Euphorbiaceae* and *Rosaceae*. While agricultural plants were represented by 15 species classified under the family *Cucurbitaceae*, *Brassicaceae Poaceae, Malvaceae*, *Apiaceae*, *Moringaceae*, *Solanaceae* (Additional file 1: Table S1).

### Analyses of pollen types

In the present study, the study area consists of mixed vegetation with multi-floral honey samples. Analysis of pollen count revealed that some plant species were more frequently represented in the honey sample. This is due to their readily available nectar coming from longer flowering periods for the particular plant species in available in the studied area. During the study period, the secondary pollen type (16–45%) was dominated by the families *Fabaceae*, *Asteraceae* and *Myrtaceae*. Other family identified under the secondary pollen types were *Poaceae*, *Apiaceae*, *Arecaceae*, *Betulaceae*, *Brassicaceae*, *Caricaceae*, *Combretaceae*, *Cucurbitaceae*, *Cyperaceae*, *Datiscaceae*, *Euphorbiaceae, Lythraceae*, *Malvaceae*, *Moringaceae, Musaceae* and *Rubiaceae*. Plant species identified under the important minor pollen types (3–15%) were mostly represented by family Fabaceae, *Asteraceae*, *Malvaceae*, *Myrtaceae*, *Cucurbitaceae*, *Euphorbiaceae*, *Rubiaceae*, *Brassicaceae*, *Combretaceae*, *Lamiaceae*, *Poaceae*, *Rosaceae*, *Solanaceae* and *Verbenaceae*. While minor pollen types (<3%) were dominated by the family *Fabaceae* followed by *Asteraceae*, *Malvaceae*, *Myrtaceae*, *Cucurbitaceae* and *Euphorbiaceae* and *Rubiaceae* (Additional file 1: Table S1). Other detected families under minor pollen types were represented by ≤3 plant species. Plant species identified under all the pollen types is shown in Additional file 1: Table S1.

### Identification of plant species using NGS technology

Present analysis detected 52 and 30 contigs from Aizawl and Champhai district respectively (Table 3). Based upon the NGS study, a total of 73 plant species were identified in two composite honey metagenome from two different districts of Mizoram, North-East India (Tables 4, 5). It was found that all the three genes used during NGS study (rbcL, matK, and ITS2) were the important marker for identification of plant species. A total of 16 plants were identified using *rbcL* gene, 29 species using matK and 29 species using ITS2 gene sequences.

**Table 3:** Sequence characteristics of the composite honey samples

Sequence characteristics	Aizawl dt.	Champhai dt.
Kmer	123	127
Number of contigs	43	30
Assemply length	32,855	16,832
N50 contigs	16	12
N50 length	673 bp	625 bp
Min contig length	281	389
Max contig length	3818	920
Avg contig length	631	621

**Table 4:** Plant species found in the composite honey sample of Aizawl using NGS technology

S. no.	Name of the plant	Family	*rbcl*	*matK*	ITS2
1	*Actinidia* sps.	*Actinidiaceae*	−	−	+
2	*Actinidia chinensis*	*Actinidiaceae*	−	−	+
3	*Anaphalis nepalensis*	*Asteraceae*	−	−	+
4	*Arachis hypogaea*	*Fabaceae*	−	−	+
5	*Augusta* sps.	*Rubiaceae*	+	−	−
6	*Barringtonia asiatica*	*Lecythidaceae*	−	−	+
7	*Betula pendula*	*Betulaceae*	−	−	+
8	*Brassica napus*	*Brassicaceae*	−	−	+
9	*Capsicum annum*	*Solanaceae*	−	−	+
10	*Ceiba pentandra*	*Malvaceae*	−	+	−
11	*Celastrus strigillosus*	*Celestraceae*	−	+	−
12	*Cicer arietinum*	*Fabaceae*	−	−	+
13	*Citrus maxima*	*Rutaceae*	+	−	−
14	*Coffea arabica*	*Rubiaceae*	−	+	−
15	*Cordia dentate*	*Boraginaceae*	+	−	−
16	*Cordia myxa*	*Boraginaceae*	−	+	−
17	*Coriandrum sativum*	*Apiaceae*	−	−	+
18	*Corymbia torelliana*	*Myrtaceae*	−	−	+
19	*Entada rheedei*	*Fabaceae*	−	+	−
20	*Erythrina subumbrans*	*Fabaceae*	−	−	+
21	*Exbucklandia populnea*	*Hamamelidaceae*	−	−	+
22	*Helianthus eggertii*	*Asteraceae*	−	+	−
23	*Lecomtella madagascariensis*	*Poaceae*	+	−	−
24	*Leucaena leucocephala*	*Fabaceae*	−	+	−
25	*Mikania micrantha*	*Asteraceae*	−	+	−
26	*Pyrus pyrifolia*	*Rosaceae*	+	−	−
27	*Pyrus ussuriensis*	*Rosaceae*	−	+	−
28	*Quercus gilva*	*Fagaceae*	−	−	+
29	*Rhoiptelea chiliantha*	*Juglandaceae*	+	−	−
30	*Salacia typhina*	*Celastraceae*	−	+	−
31	*Smilax* sps.	*Smilacaceae*	−	+	−
32	*Solanum carolinense*	*Solanaceae*	−	−	+
33	*Solanum lycopersicum*	*Solanaceae*	−	−	+
34	*Solanum pennellii*	*Solanaceae*	−	−	+
35	*Sophora tetraptera*	*Fabaceae*	−	−	+
36	*Trema tomentosa*	*Cannabaceae*	−	+	−
37	*Tripterygium regelii*	*Celestraceae*	−	+	−
38	*Triticum dicoccon*	*Poaceae*	+	−	−
39	*Ugni molinae*	*Myrtaceae*	−	+	−
40	*Vitis vinifera*	*Vitaceae*	−	−	+
41	*Wahlenbergia augustifolia*	*Campanulaceae*	−	+	−
42	*Wahlenbergia gloriosa*	*Campanulaceae*	+	−	−
43	*Wendlandia tinctoria*	*Rubiaceae*	−	−	+

**Table 5:** Plant species found in the composite honey sample of Champhai using NGS technology

S. no.	Name of the plant	Family	*rbcL*	*matK*	ITS2
1	*Ageratina adenophora*	*Asteraceae*	−	−	+
2	*Ageratum conyzoides*	*Asteraceae*	+	−	−
3	*Ageratum houstonianum*	*Asteraceae*	−	+	−
4	*Alangium chinense*	*Cornaceae*	−	+	−
5	*Albizia procera*	*Fabaceae*	−	−	+
6	*Archidendron hirsutum*	*Fabaceae*	+	−	−
7	*Arenga pinnata*	*Arecaceae*	+	−	−
8	*Bambusa oldhamii*	*Poaceae*	−	−	+
9	*Bischofia javanica*	*Phyllanthaceae*	+	−	−
10	*Calamus caryotoides*	*Arecaceae*	−	−	+
11	*Calamus castaneus*	*Arecaceae*	−	+	−
12	*Callistemon citrinus*	*Myrtaceae*	+	−	−
13	*Carya hunanensis*	*Juglandaceae*	−	+	−
14	*Coffea arabica*	*Rubiaceae*	−	+	−
15	*Dimocarpus longan*	*Sapindaceae*	+	−	−
16	*Eucalyptus melliodora*	*Myrtaceae*	−	−	+
17	*Helianthus maximiliani*	*Asteraceae*	−	+	−
18	*Juglans olanchana*	*Juglandaceae*	−	+	−
19	*Juglans regia*	*Juglandaceae*	−	+	−
20	*Mikania micrantha*	*Asterceae*	−	−	+
21	*Paneroa stachyofolia*	*Asterceae*	−	+	−
22	*Prunus cerasifera*	*Rosaceae*	−	+	−
23	*Psidium guajava*	*Myrtaceae*	−	+	−
24	*Schima wallichii*	*Theaceae*	−	+	−
25	*Sorghum halepense*	*Poaceae*	+	−	−
26	*Tetrameles nudiflora*	*Tetramelaceae*	−	+	−
27	*Thysanolaena latifolia*	*Poaceae*	−	−	+
28	*Trema* sps.	*Cannabiaceae*	+	−	−
29	*Wendlandia formosana*	*Rubiaceae*	−	+	−
30	*Zanthoxylum piperitum*	*Rutaceae*	−	−	+

**Fig. 2:**
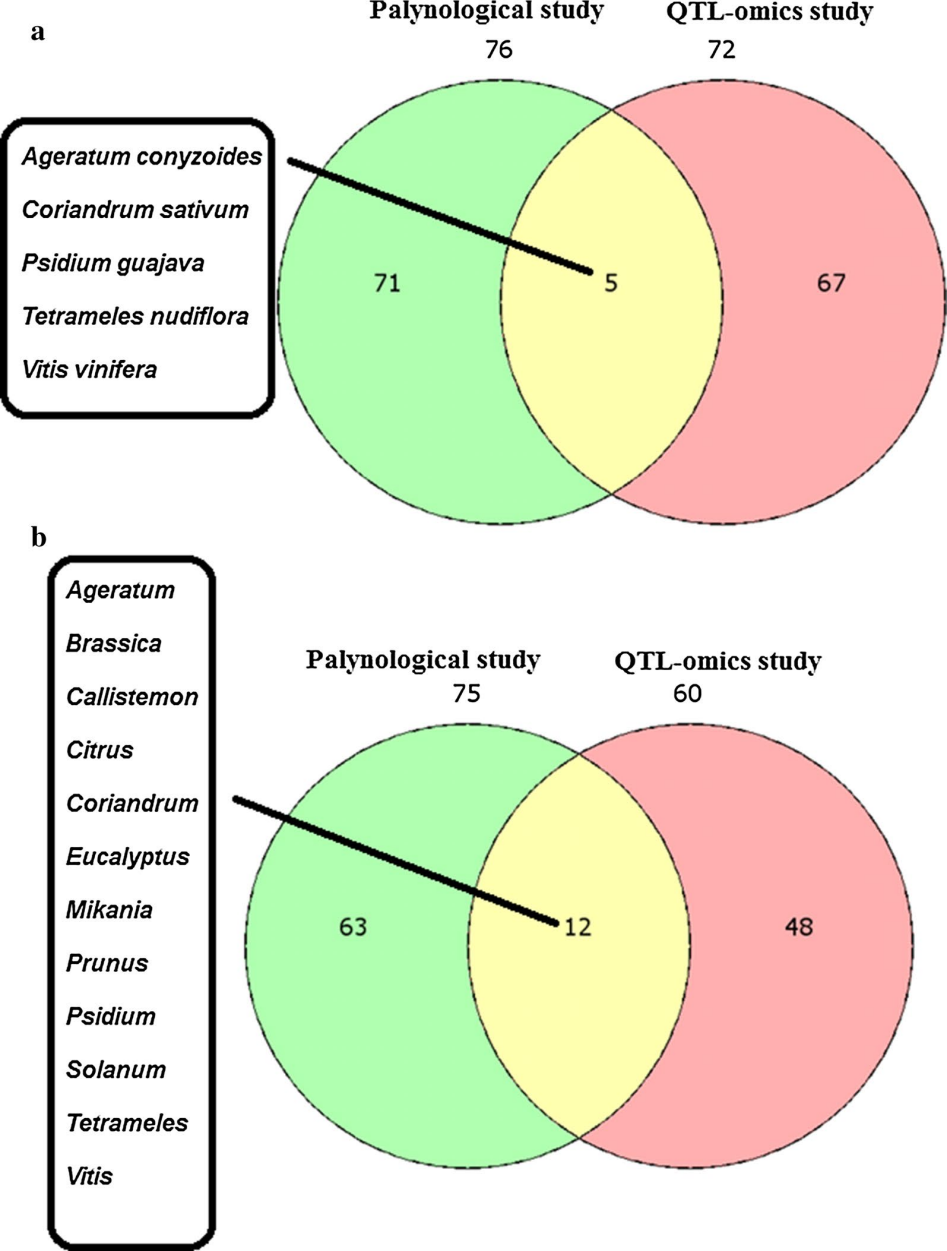
Unique and shared plant species identified in palynological and QTLomics in **a** Aizawl district. **b** Champhai district of Mizoram, Northeast India

At the species level, only five plant species were found to be the common in both palynological and NGS studies, whereas 12 common genera were identified in both the approaches. This might be due to the inadequate information on gene information, which hinders the identification of all the polleniferous plant species using NGS approach (Fig. 2a, b). The only species *Coffea arabica* was found to be commonly present in both Champhai and Aizawl district.

## Discussion

Next generation sequencing (NGS) has been successfully used for taxonomic assessment of polleniferous plant from honey samples (Richardson et al. 2015a, b; Sickel et al. 2015; Keller et al. 2015). Present study combines traditional microscopic analysis with DNA metabarcoding to understand the scope of identifying the polleniferous plants from honey samples.

The two approach for study DNA metabarcoding identified 74 number of polliniferous plant species from the two districts of the state Mizoram, while melissopalynological study identified 76 numbers of plant species. Hence, the two techniques are important and relevant for identifying the polliniferous plants. Rechardson et al. (2015b) studied honey pollen samples using three metabarcoding targeting (ITS2, *matK*, and *rbcL*) as well as by light microscopy and found a significant correlation between the relative abundance of the pollen types in the studied samples with both metabarcoding and microscopic observation (Richardson et al. 2015b). They also denoted that multilocus metabarcoding is more reliable than single-locus analyses (Sickel et al. 2015).

Mizoram falling under the Indo-Burma Biodiversity hotspot zone is possessing large forest coverage. Due to Jhum cultivation, forests in Mizoram are degraded and analyzing pollen present in honey samples will help to understand the effect of such anthropogenic activities that affect the diversity of plant species and floral resource quality. This will also help in habitat restoration and conservation efforts (Myers et al. 2000; De Mandal et al. 2016). In both the approaches, most of the plant species did not show similar taxonomic placement up to the genus level or by the species level. This might be due to the lack of databases for the polleniferous plants of the studied region or lack of sufficient information for taxonomic identification.

Melissopalynology study the microscopic analysis of pollen content of the honey from the locality, with field study involving phenology provide reliable information regarding the floral types which serve as the pollen sources for the honey bees. Pollen found in honey is used to determine the honey types, quality control and to ascertain whether honey is adulterated or not (Villanueva 1994). From the pollen spectra, it was observed that the two districts include both naturalized flora as well as cultivated crops. It also gives a wider knowledge of bee preferences in local floral. Generally, entomophilic plants were numerous in the pollen spectrum of each honey sample studied and the honey from the source localities was fairly rich in pollen types. The microscopical analysis of honey is important in establishing the seasonal pollen spectra of honey from various climatic and geographical areas, for evaluation of honey originated from various physiographic region (Chaturvedi 1983). In the present study, many pollens were unidentifiable, which reflects the drawbacks in the taxonomical classification system.

The outcome of this study depicts the link between honey bees and its foraging plant species, honeybee foraging plant diversity using a DNA metabarcoding approach. In the present study, palynology data has identified many plant species that were not identifiable by NGS. This might be because of incomplete plant database and future research should focus on strengthening the information on the plant DNA barcode genes. In our study, majority of the pollen were unidentifiable using palynology which might represent many other plant species. These plant species might have been identified using the NGS approach. It will be of immense value for the development of beekeeping industry for the studied area and for the entire region and this information could be used to selectively grow native plants that are important for the honey bees. The present study will be helpful for identifying different floral sources used by honey bees and improved the conservation of economically viable plants.

## Additional file

**Additional file 1: Table S1.** Palynological analysis of the honey samples.

## Abbreviations

rbcL: ribulose-bisphosphate carboxylase gene
MatK: maturase K gene
ITS: internal transcribed spacer
DNA: deoxyribonucleic acid
PCR: polymerase chain reaction
NGS: next-generation sequencing
