# Correction to: Meta-barcoding in combination with palynological inference is a potent diagnostic marker for honey floral composition

**DOI:** 10.1186/s13568-017-0489-8

**Published:** 2017-10-11

**Authors:** Rama Chandra Laha, Surajit De Mandal, Lalhmanghai Ralte, Laldinfeli Ralte, Nachimuthu Senthil Kumar, Guruswami Gurusubramanian, Ramalingam Satishkumar, Raja Mugasimangalam, Nagesh Aswathnarayana Kuravadi

**Affiliations:** 1 Departments of Botany, Biotechnology and Zoology, School of Life Sciences, Mizoram University, Aizawl, Mizoram 796004 India; 2 Department of Biotechnology, Bharathiar University, Coimbatore, 641046 India; 3 Genotypic Technologies, Bangalore, India; 4 QTLomics Technologies, Bangalore, India

## Correction to: AMB Expr (2017) 7:132 DOI 10.1186/s13568-017-0429-7

In the version of this article that was originally published (Laha et al. 2017) the authors did not properly reference one paragraph in the Introduction section, specifically the paragraph:.

“While some efforts have been made to develop protocols to ascertain the entomological sources of honey (Schnell et al. 2010), most have focused on identifying its plant origin. Past studies have often relied upon diagnostic phytochemicals (Cotte et al. 2004; Tosun 2013) or the study of pollen in honey (melissopalynology) (Alves and Santos 2014). Although the latter approach requires considerable expertise and cannot distinguish many plant species (Kaškonienė and Venskutonis 2010), yet it is a powerful diagnostic tool, especially when used with other methods (Hawkins et al. 2015). However, melissopalynology is ineffective in cases where low value honey is filtered to remove its source pollen and spiked with pollen from the desired monoflora (Kaškonienė and Venskutonis 2010).”

The authors wish to acknowledge the article “Rapid identification of the botanical and entomological sources of honey using DNA metabarcoding” by Sean W.J. Prosser and Paul D.N. Hebert as reference for this paragraph (Prosser and Hebert 2017). The authors wish to apologize for this omission.
