# Supplementary material for: Meta-barcoding in combination with palynological inference is a potent diagnostic marker for honey floral composition
Source: AMB Express. 2017 Jun 24;7:132. doi: 10.1186/s13568-017-0429-7 (PMC5483214; doi:10.1186/s13568-017-0429-7)
Supplement: Supplementary file 1 — Additional file 1: Table S1. Palynological analysis of the honey samples. [file 13568_2017_429_MOESM1_ESM.docx]

**Article title**: Meta-barcoding in combination with palynological inference is a potent diagnostic marker for honey floral composition

**Journal name**: AMB Express

**Author names**:

Rama Chandra Laha^1☯^*, Surajit De Mandal^1☯^, Lalhmanghai Ralte^1^, Laldinfeli Ralte^1^, Nachimuthu Senthil Kumar^1^, Guruswami Gurusubramanian^1^, Ramalingam Satishkumar^2^, Raja Mugasimangalam^3^, Nagesh Aswathnarayana Kuravadi^4^

**Affiliation:**

^1^Departments of Botany, Biotechnology and Zoology, School of Life Sciences, Mizoram University, Aizawl-796004, Mizoram, India. ^2^Department of Biotechnology, Bharathiar University, Coimbatore – 641 046, India. ^3^Genotypic Technologies, Bangalore, India. ^4^QTLomics Technologies, Bangalore, India.

**e-mail address of the corresponding author**: rc_laha@yahoo.com

**Supplementary Table S1: Palynological analysis of the honey samples.**

| **S.No** | **Family** | **Species** | **Vg** | **A1** | **A2** | **A3** | **A4** | **A5** | **A6** | **A7** | **A8** | **A9** | **A10** | **C1** | **C2** | **C3** | **C4** | **C5** | **C6** | **C7** | **C8** | **C9** | **C10** |
| --- | --- | --- | --- | --- | --- | --- | --- | --- | --- | --- | --- | --- | --- | --- | --- | --- | --- | --- | --- | --- | --- | --- | --- |
| 1 | ***Amaranthaceae*** | *Amaranthus sps.* | WP | - | I | - | - | - | - | M | - | - | - | - | I | I | I | - | - | - | - | - | M |
| 2 | ***Anacardaceae*** | *Mangifera indica* | HP | - | M | - | I | - | - | - | - | - | - | M | - | - | - | I | I | - | - | M | - |
| 3 | ***Apiaceae*** | *Coriandrum sativum* | AP | - | - | - | S | - | - | - | I | - | - | I | - | - | - | S | S | I | - | - | S |
| 4 | ***Arecaceae*** | *Cocos nucifera* | HP | M | S | M | - | I | - | - | I | - | - | M | - | I | M | S | - | - | - | I | - |
| 5 | ***Asclepiadaceae*** | *Asclepias curassavica* | OP | - | - | - | I | - | - | - | I | - | - | M | - | - | - | - | - | M | - | - | I |
| 6 | ***Asteraceae*** | *Ageratum conyzoides* | WP | - | S | - | M | - | S | - | I | - | I | - | - | S | M | - | - | - | I | - | -M |
| 7 |  | *Biens pilosa* | WP | - | - | M | - | - | - | M | - | - | - | I | - | M | - | - | - | - | I | - | M |
| 8 |  | *Cosmos sulphureus* | WP | - | - | - | I | M | - | - | - | M | - | I | - | - | M | I | - | - | - | I | - |
| 9 |  | *Matricaria chomonulla* | WP | - | - | - | - | - | M | - | - | - | - | - | - | - | - | - | M | - | - | - | - |
| 10 |  | *Mikania micranta* | WP | S | - | S | - | - | M | - | - | S | S | - | S | - | - | M | - | - | M | - | I |
| 11 |  | *Spilanthes acmella* | WP | - | - | S | - | - | I | I | - | - | - | I | - | - | - | - | - | S | M | - | - |
| 12 |  | *Tagetes erecta* | WP | M | - | I | - | I | - | M | - | M | - | - | M | - | - | - | - | M | - | - | I |
| 13 |  | *Tithonia diversifolia* | WP | - | M | - | M | - | I | - | M | - | I | - | M | - | M | - | - | I | - | I | - |
| 14 |  | *Zinnia elgans* | WP | M | - | I | - | I | - | M | - | M | - | - | M | - | - | - | - | M | - | - | I |
| 15 | ***Betulaceae*** | *Alnus nitida* | WP | I | - | - | I | M | I | - | M | - | S | - | - | - | - | I | I | - | - | S | S |
| 16 | ***Bignoniaceae*** | *Tecoma stans* | WP | - | - | - | - | M | - | - | - | - | I | - | - | I | - | I | - | - | M | - | - |
| 17 | ***Bombacaceae*** | *Bombax ceiba* | WP | - | - | S | - | I | M | - | - | S | S | - | - | M | - | - | - | S | I | - | M |
| 18 | ***Brassicaceae*** | *Brassica campestris* | AP | I | - | - | S | - | - | S | S | - | - | S | - | - | I | - | M | I | - | M | M |
| 19 |  | *Raphanus sativus* | AP | - | - | I | I | - | - | - | - | - | - | - | - | - | - | M | I | - | - | - | - |
| 20 | ***Caricaceae*** | *Carica papaya* | HP | M | I | - | M | - | - | - | S | - | - | - | - | - | - | S | - | M | - | - | S |
| 21 | ***Combretaceae*** | *Terminalia crenulata* | WP | - | - | - | - | - | - | - | M |  | - | I | - | - | - | - | - | - | - | - | - |
| 22 |  | *Terminalia bellirica* | WP | S | I | - | S | - | I | M | - | M | - | S | M | M | I | S | - | I | S | M | I |
| 23 | ***Cucurbitaceae*** | *Cucumis sativus* | AP | M | M | I | - | S | M | I | S | - | M | M | - | I | - | - | S | S | - | - | - |
| 24 |  | *Cucurbita pepo* | AP | - | - | M | - | - | I | - | - | - | - | M | - | - | - | M | - | - | - | - | I |
| 25 |  | *Momordica charantia* | AP | M | - | - | - | I | - | M | - | - | I | M | - | I | - | - | M | - | - | - | I |
| 26 |  | *Sechium edule* | AP | - | - | I | M | - | - | - | M | - | - | - | M | M | - | - | - | I | - | - | M |
| 27 | ***Cyperaceae*** | *Cyperus rotundus* | WP | M | - | - | - | M | - | S | - | I | M | - | M | - | M | - | I | - | - | M | - |
| 28 | ***Datiscaceae*** | *Tetrameles nudiflora* | WP | - | M | - | - | - | - | S | - | - | - | - | - | M | - | - | M | S | M | I | S |
| 29 | ***Elaeocarpaceae*** | *Elaeocarpus lanceifolius* | WP | - | - | M | - | - | I | - | - | - | I | - | - | - | - | M | - | - | M | - | - |
| 30 | ***Euphorbiaceae*** | *Croton jaufra* | WP | I | - | I | M | - | M | - | - | - | - | - | M | M | - | - | M | - | M | - | - |
| 31 |  | *Emblica officianalis* | WP | - | - | S | - | - | I | - | I | M | - | - | - | - | M | I | - | S | - | - | I |
| 32 |  | *Euphorbia pulcherrima* | OP | - | - | - | - | M | - | - | - | - | M | I | - | - | - | - | - | - | - | I | - |
| 33 |  | *Riccinus communis* | WP | M | - | - | I | - | - | I | - | - | M | - | M | - | - | I | M | - | - | M | - |
| 34 | ***Fabaceae*** | *Acacia pruinescens* | WP | M | - | M | - | S | M | - | S | M | M | - | M | M | M | - | - | - | I | I | M |
| 35 |  | *Bauhinia variegata* | WP | - | M | - | M | - | - | I | - | - | I | - | - | I | - | M | - | - | I | - | - |
| 36 |  | *Caesalpinia pulcherrima* | WP | M | - | - | - | - | - | M | - | - | - | I | - | - | I | - | - | I | M | M | - |
| 37 |  | *Cassia javanica* | WP | - | - | I | - | - | I | - | - | I | - | - | - | I | - | - | - | I | - | - | M |
| 38 |  | *Derris robusta* | WP | - | - | - | - | M | - | - | M | - | I | I | M | - | I | - | M | - | - | - | I |
| 39 |  | *Mimosa pudica* | WP | - | M | - | - | M | - | - | - | M | - | - | M | - | M | - | I | - | - | M | M |
| 40 |  | *Parkia timoriana* | WP | - | S | - | - | - | M | - | M | - | - | M | - | - | I | - | - | - | I | S | - |
| 41 |  | *Phaseolus vulgaris* | AP | - | - | - | M | - | - | M | - | - | - | - | - | M | - | I | - | M | - | I | - |
| 42 |  | *Pisum sativum* | AP | M | - | - | - | S | S | - | M | - | M | - | I | - | S | - | - | M | - | I | - |
| 43 |  | *Tamarindus indica* | WP | M | - | I | I | I | - | I | - | M | S | - | - | S | I | - | M | - | - | M | - |
| 44 | ***Fagaceae*** | *Castanopsis tribuloides* | WP | M | - | - | I | - | - | - | M | - | - | - | M | - | - | - | - | M | - | - | - |
| 45 | ***Lamiaceae*** | *Holmskioldia sanguine* | WP | - | - | M | - | - | - | - | - | M | - | M | M | - | I | - | - | - | M | - | - |
| 46 |  | *Leucosceptrum canum* | WP | - | M | - | - | I | - | - | - | M | - | M | - | M | - | I | - | - | M | - | I |
| 47 | ***Lythraceae*** | *Lagerstromia speciosa* | WP | - | S | - | - | - | S | - | - | M | - | - | M | M | - | M | - | M | - | - | M |
| 48 |  | *Punica granatum* | HP | - | - | - | M | - | - | M | - | - | I | - | - | - | - | - | - | - | M | M | I |
| 49 | ***Malvaceae*** | *Althaea rosea* | WP | - | M | - | M | - | - | - | M | - | M | - | - | - | I | - | I | M | - | M | - |
| 50 |  | *Anthurium andreanum* | OP | M | M | - | I | - | - | M | - | I | - | M | - | - | - | M | - | M | - | M | - |
| 51 |  | *Hibiscus rosasinenensis* | OP | - | - | M | - | - | M | - | - | - | I | - | - | I | - | M | - | M | M | - | M |
| 52 |  | *Ipomoea batatus* | AP | - | M | - | - | I | - | - | I | - | - | M | - | - | S | - | I | - | - | - | I |
| 53 |  | *Malvaviscus arboreus* | WP | - | - | - | M | - | - | - | - | - | - | - | - | - | M | I | M | - | - | - | M |
| 54 | ***Moringaceae*** | *Moringa oleifera* | AP | - | S | - | - | - | - | - | M | M | M | S | - | - | S | M | - | - | M | - | I |
| 55 | ***Musaceae*** | *Musa paradisiaca* | HP | M | - | M | I | - | - | M | M | - | - | - | S | M | - | - | I | - | M | - | M |
| 56 | ***Myrtaceae*** | *Callistemon lanceolatus* | WP | - | - | - | - | - | - | I | - | - | - | M | - | - | I | - | - | M | - | - | - |
| 57 |  | *Eucalyptus tereticornis* | WP | - | S | - | - | M | - | I | - | - | - | - | - | S | - | - | M | S | - | S | M |
| 58 |  | *Psidium guajava* | HP | S | - | I | - | I | M | M | - | I | M | S | - | M | - | M | M | - | M | - | - |
| 59 |  | *Syzgium cumini* | HP | S | - | - | M | M | - | I | - | I | - | I | - | - | M | S | - | I | - | I | M |
| 60 |  | *Syzygium jambos* | HP | - | - | - | - | - | M | - | - | - | - | - | I | - | - | - | M | - | I | M | - |
| 61 | ***Oxilidaceae*** | *Averrhoa carambola* | WP | I | - | I | - | - | - | M | - | - | - | - | - | I | - | - | M | - | - | M | I |
| 62 | ***Poaceae*** | *Oryza sativa* | AP | M | M | - | S | M | - | S | - | - | S | - | M | - | M | - | S | I | S | M | M |
| 63 |  | *Zea mays* | AP | - | I | S | - | S | M | - | S | I | - | M | - | S | - | M | - | I | - | - | S |
| 64 | ***Polygonaceae*** | *Antigonon leptopus* | WP | - | - | M | - | - | M | - | I | M | - | - | M | - | - | M | - | I | - | M | - |
| 65 | ***Rosaceae*** | *Prunus persica* | WP | - | - | M | - | I | - | M | - | - | M | - | M | - | - | - | M | - | - | - | M |
| 66 |  | *Rosa macrophylla* | OP | M | - | I | - | M | - | - | M |  | M | - | M | - | I | - | - | M | - | M | - |
| 67 | ***Rubiaceae*** | *Coffee Arabica* | HP | - | - | - | S | - | M | S | - | S | S | S | - | M | - | I | - | - | I | - | I |
| 68 |  | *Ixora coccinea* | OP | M | - | - | - | - | M | - | - | - | - | - | - | - | M |  | M | - | I | - | - |
| 69 |  | *Jatropha curcus* | WP | - | M | - | - | M | - | - | - | M | - | - | - | I | - | I | - | - | M | I | M |
| 70 | ***Rutaceae*** | *Citrus limon* | HP | M | - | I | - | M | I | - | - | M | - | - | M | - | I | - | M | M | - | M | - |
| 71 | ***Solanaceae*** | *Nicotianum tobaccum* | WP | - | M | - | - | M | M | I | - | I | M | M | I | - | - | - | M | - | - | M | M |
| 72 |  | *Solanum melongena* | AP | - | - | - | M | - | I | - | M | - | M | - | M | - | M | M | - | M | I | M | - |
| 73 | ***Tropaeolaceae*** | *Tropaelum majus* | WP | - | - | - | - | - | - | - | M | - | - | M | - | M | M | - | I | - | - | - | M |
| 74 | ***Verbenaceae*** | *Lantana camara* | WP | M | - | - | M | M | - | M | - | - | - | - | - | - | M | - | - | I | - | M | - |
| 75 |  | *Callicarpa arborea* | WP | - | - | M | - | - | M | - | I | - | - | - | - | M | - | - | M | - | I | - | - |
| 76 | ***Vitaceae*** | *Vitis vinifera* | HP | - | - | M | - | - | M | - | - | - | - | - | M | M | - | M | M | - | I | - | M |

Vg- Vegetation; WP-Wild plant; HP- Horticultural plant; OP- Ornamental plant; AP- Agricultural plant, I- Important minor pollen; M- Minor pollen; S- Secondary dominant pollen
